# Effects of systemic glycine on accumbal glycine and dopamine levels and ethanol intake in male Wistar rats

**DOI:** 10.1007/s00702-020-02284-x

**Published:** 2020-12-22

**Authors:** Yasmin Olsson, Helga Höifödt Lidö, Klara Danielsson, Mia Ericson, Bo Söderpalm

**Affiliations:** 1 Addiction Biology Unit, Department of Psychiatry and Neurochemistry, Institute of Neuroscience and Physiology, The Sahlgrenska Academy, University of Gothenburg, PO Box 410, 405 30 Gothenburg, Sweden; 2 Beroendekliniken, Sahlgrenska University Hospital, Gothenburg, Sweden; 3 Department of Neurology, Sahlgrenska University Hospital, Gothenburg, Sweden

**Keywords:** Dopamine, Alcohol consumption, In vivo microdialysis, Glycine receptor, Nucleus accumbens

## Abstract

Approved medications for alcohol use disorder (AUD) display modest effect sizes. Pharmacotherapy aimed at the mechanism(s) by which ethanol activates the dopamine reward pathway may offer improved outcomes. Basal and ethanol-induced accumbal dopamine release in the rat involve glycine receptors (GlyR) in the nucleus accumbens (nAc). Glycine transporter 1 (GlyT-1) inhibitors, which raise extracellular glycine levels, have repeatedly been shown to decrease ethanol intake in the rat. To further explore the rational for elevating glycine levels in the treatment of AUD, this study examined accumbal extracellular glycine and dopamine levels and voluntary ethanol intake and preference in the rat, after systemic treatment with glycine. The effects of three different doses of glycine i.p. on accumbal glycine and dopamine levels were examined using in vivo microdialysis in Wistar rats. In addition, the effects of the intermediate dose of glycine on voluntary ethanol intake and preference were examined in a limited access two-bottle ethanol/water model in the rat. Systemic glycine treatment increased accumbal glycine levels in a dose-related manner, whereas accumbal dopamine levels were elevated in a subpopulation of animals, defined as dopamine responders. Ethanol intake and preference decreased after systemic glycine treatment. These results give further support to the concept of elevating central glycine levels to reduce ethanol intake and indicate that targeting the glycinergic system may represent a pharmacologic treatment principle for AUD.

## Introduction

Ethanol consumption contributes substantially to the global burden of disease (WHO 2018), and individuals with alcohol use disorder (AUD) are especially prone to high ethanol consumption. AUD thus generates enormous socioeconomic costs encompassing both healthcare expenses and social harm (Rehm et al. 2009). One way of combating AUD is through pharmacotherapy, and current approved medications are disulfiram, acamprosate, naltrexone and nalmefene. Disulfiram has a good short-term effect but presupposes complete abstinence from ethanol, since its aversive effect stems from accumulation of toxic acetaldehyde when consuming ethanol. The latter three compounds are believed to target ethanol intake by interacting with ethanol reward and craving. However, since effect sizes of current medications are low (Cohen’s *d* = 0.2) (Jonas et al. 2014), improved pharmacotherapies are warranted.

In similarity to other drugs of abuse, ethanol’s reinforcing properties involve enhanced dopamine activity in the nucleus accumbens (nAc), an important part of the mesolimbic dopamine system (Di Chiara and Imperato 1988; Gonzales et al. 2004; Spanagel 2009). A large body of evidence suggests that transition from recreational to compulsive drinking, characteristic of AUD, involves neuroadaptations in the mesolimbic reward system (Gonzales et al. 2004; Koob and Volkow 2010). Pharmacotherapies tailored to act directly on the mechanism(s) by which ethanol interferes with the reward system could offer an improved treatment outcome. Our research group has in several studies obtained data indicating that ethanol targets nAc glycine receptors (GlyR) that probably inhibit γ-aminobutyric acid (GABA)-ergic projections to the ventral tegmental area (VTA), ultimately leading to increased dopamine release in the nAc cf. (Molander and Söderpalm 2005b; Soderpalm et al. 2017). Indeed, recent studies have confirmed that the function of non-synaptic GlyR located on accumbal GABAergic projection neurons are potentiated by ethanol (Forstera et al. 2017). Hence, modulation of CNS GlyR has discerned as a promising opportunity for interfering with the reinforcing properties of ethanol.

One way to modulate GlyR is to raise extracellular levels of glycine. The principal regulator of CNS glycine levels is the glycine transporter 1 protein (GlyT-1) (Betz et al. 2006). Interestingly, the GlyT-1-inhibitor Org 25935 increases basal glycine and dopamine levels in the rat nAc and attenuates the ethanol-induced dopamine elevation in the same region for a subpopulation of animals previously denoted as responders (i.e., rats presenting elevated dopamine levels upon local treatment with glycine (in the nAc) or systemic treatment with Org 25935) (Höifödt Lidö et al. 2011; Lidö et al. 2009; Molander et al. 2005; Molander and Soderpalm 2005a). Further, systemic GlyT-1-inhibitors robustly and dose-dependently decrease ethanol intake and preference in the rat with no signs of tolerance development, in contrast to e.g., acamprosate (Molander et al. 2007; Vengeliene et al. 2010, 2018; Lidö et al. 2012; Chau et al. 2018).

This strong body of evidence created the imperative for a clinical trial examining whether Org 25935 prevents relapse in detoxified AUD patients. This study was, however, interrupted pre-term when an interim analysis indicated futility to show a difference between active treatment and placebo (de Bejczy et al. 2014). Several shortcomings in the study design could explain this unexpected failure. For example, a benzodiazepine taper preceded the study, possibly contributing to a profound placebo effect leaving little room for the drug to manifest improved effect (de Bejczy et al. 2014). The very low drinking levels when initiating treatment with Org 25935 in essence prevented a sustained interaction between ethanol and the drug, that may be required to gradually reduce ethanol intake cf. (Soderpalm et al. 2017). Moreover, the lack of an objective marker for ethanol intake, e.g., B-PEth, may have occluded a true drug effect (de Bejczy et al. 2015; Walther et al. 2015).

In demur to hastily dismiss the glycinergic treatment concept and in light of a strong, preclinical rational, we wanted to further explore the concept of raising brain glycine levels by applying a non-patented glycinergic compound, i.e., glycine itself. High-dose glycine has been demonstrated to be safe and efficacious in the human setting, when tried as an adjunctive treatment for negative symptoms of schizophrenia (Harvey and Yee 2013; Heresco-Levy et al. 1999). This is the first study to examine the effect of systemic glycine on accumbal glycine and dopamine levels and on ethanol intake in the rat. Previously, dopamine elevations in responding animals and decreased ethanol intake were achieved when Org 25935 increased peak levels of accumbal glycine by 85–100%, as measured by in vivo microdialysis (Lidö et al. 2009; Molander et al. 2007). Hence, here we aimed at finding a dose of systemic glycine, which would increase extracellular glycine and dopamine levels in the nAc in the rat to a similar extent as Org 25935 and then examine the effect of this dose on ethanol intake and preference in a limited access two-bottle ethanol/water model in the rat.

## Materials and methods

### Animals

For in vivo microdialysis, a total number of 75 male Wistar rats weighing 260–280 g at arrival were supplied by Envigo (Netherlands) and Janvier (France). Animals were housed four to a cage (55 × 35 × 20 cm) under controlled environmental conditions comprising constant room temperature of 22 °C, humidity of 65% and regular light–dark conditions with lights on at 07:00 a.m. and off at 07:00 p.m. For the ethanol consumption test, 50 male Wistar rats weighing 160–180 g at arrival were obtained from Envigo (Netherlands). Animals were single-housed (40 × 24 × 18 cm) at room temperature of 22 °C, humidity of 65% and kept under reversed light–dark conditions with lights on at 10:00 p.m. and off at 10:00 a.m. All animals had free access to tap water and standard rat feed (Harlan Teklad, Norfolk, England) and were acclimatized to the facility for at least 1 week before any procedures were initiated. The study was performed in accordance with protocols approved by the Ethics Committee for Animal Experiments, Gothenburg, Sweden.

### Drugs and solutions

Glycine (Sigma-Aldrich, Sweden) was dissolved in 0.9% NaCl solution to a desired dose of 200 mg/kg, 400 mg/kg or 800 mg/kg and administered i.p. in a volume of 2.0 ml/kg. Ethanol (95%; Kemetyl AB, Sweden) was diluted in tap water and administered to the animals in the home cage fluid bottles.

### Surgical procedure

Animals were anesthetized with isoflurane (Baxter Medical AB, Kista, Sweden) and mounted onto a stereotactic instrument (David Kopf Instruments, AgnTho’s AB, Sweden). A heating pad was used to prevent hypothermia during surgery. Three holes were drilled for the placement of an I-shaped, custom made probe with a semi-permeable membrane and two anchoring screws. The probe was lowered mono-laterally into the nAc core–shell borderline region (A/P: + 1.85, M/L: − 1.4, D/V: − 7.8 mm relative to bregma and dura, coordinates from Paxinos and Watson 2007. The core–shell border region was targeted since this subregion of the nAc is associated with dopamine elevation upon ethanol intake (Howard et al. 2009). The active space of the probe was 2 mm and refers to the exposed length of the membrane, where the fluid exchange between perfused and extracellular fluid take place. Probes and anchoring screws were fixed to the skull using Harvard cement (DAB Dental AB, Stockholm, Sweden). Marcain^®^ (buvipacaine, AstraZeneca, Sweden) was applied locally for analgesic purposes and 2 ml NaCl 0.9% was injected subcutaneously to prevent dehydration. Before initiation of in vivo microdialysis, animals were placed in individual cages and allowed to recover for 48 h.

### Microdialysis, experimental procedure

**Fig. 1:**
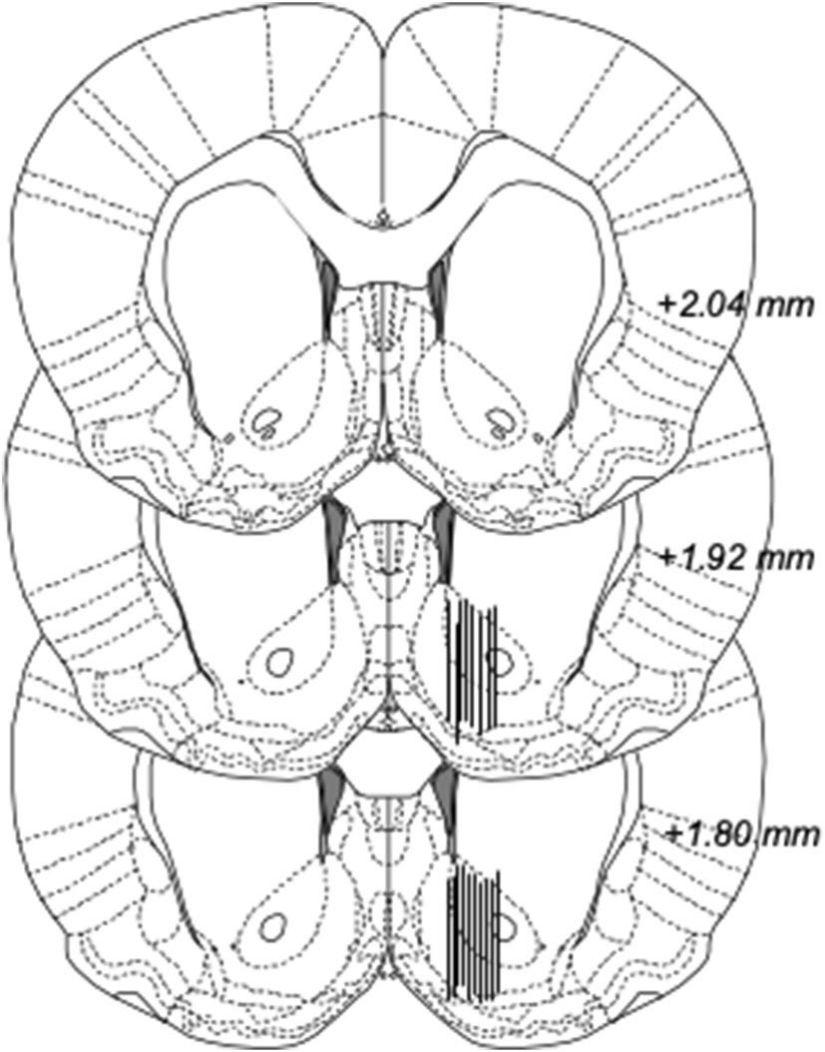
Probe placement verification. Location of 18 representative microdialysis probes in the nAc, as depicted by black lines. The active space of the probe (2 mm of its inferior aspect) targets the nAc core–shell border region. Numbers on the right side of the figure represent distance from bregma

On the day of the experiment, the sealed inlet and outlet of the probe was cut open and connected to a microperfusion pump (U-864 Syringe Pump, AgnTho’s, Sweden) via a swivel allowing the animal to move freely in the cage. The probe was perfused with Ringer solution at a rate of 2 μl/min for 2 h prior to sampling to achieve equilibrium around the active space of the probe. Subsequently, four baseline samples were collected and animals with dopamine levels differing more than 10% were excluded. Rats were randomly assigned to receive either glycine in three different doses (200, 400 and 800 mg/kg) or vehicle administered i.p. at time point 0. Following drug administration, a total number of 6 dialysate samples were collected. Sampling throughout the experiment took place every 20 min, the volume for each sample comprised of approximately 40 μl extracellular fluid dialysate. At the end of the experiment, rats were euthanized, and brains were placed in accustain (Sigma diagnostics, USA) for 3–6 days before probe placement was verified with the naked eye using a vibroslicer (Campden Instruments Ltd., Lafayette, IN, USA). Animals with incorrect probe placement and/or substantial hemorrhage around the probe were excluded. Probe placement is illustrated in Fig. 1. Microdialysate dopamine content was separated and quantified using high performance liquid chromatography (HPLC) with an electrochemical cell detector as previously described (Ulenius et al. 2019). An external standard containing 3.25 nM of dopamine was used to identify the dopamine peak and to quantify dopamine concentrations in the dialysates. All samples of dopamine were analyzed on-line and the remainder of the dialysate was preserved in sodium azide (NaN_3_) and stored at a cold temperature for later analysis of glycine in a different HPLC set up with fluorescence detection. External standards containing 500 nM and 1000 nM of glycine were used for glycine analysis. Baseline levels for each animal was set to 100%.

### Screening for ethanol preference

The animals were presented with intermittent access to ethanol 3 sessions per week. The bottles were added concurrent with the beginning of the dark period and removed after 24 h. For the first five sessions, the animals had access to 6% (v/v) ethanol in addition to water, and to 12% ethanol for the remaining experiment. Previous observations from our laboratory using Wistar rats indicate that ethanol consumption is maximal approximately at these concentrations that therefore have been applied in our previous ethanol consumption studies (Söderpalm et al. 2019). Water and ethanol intake were monitored intermittently for seven weeks. Ethanol preference was calculated as liquid consumed (%) from the ethanol bottle in relation to the total fluid intake. The animals were supervised on a daily basis and weighed weekly (Fig. 6d).

### Voluntary ethanol consumption, experimental procedure

Following the initial screening, all animals with a minimum ethanol consumption of 1 g/kg/24 h were placed on a limited access paradigm, where the animals had access to ethanol and water for 2.5 h per day. During baseline limited access recordings (5 days) all animals received a systemic injection of vehicle 30 min prior to access to the bottles, to minimize the influence of stress from the injection on voluntary ethanol consumption. Following baseline, 28 of the animals were divided into 2 treatment groups [glycine (400 mg/kg) or vehicle] and the rats received their designated treatment 30 min prior to access to the bottles for seven days. The choice of a glycine dose of 400 mg/kg was motivated by this dose being associated with the largest proportion of dopamine responders (Fig. 4) and the highest dopamine peak (Figs. 3, 4, 5) in the microdialysis studies.

### Statistical analysis

Data are presented as mean values ± SEM. Two-way ANOVAs with repeated measures followed by Dunnet’s post hoc tests were used for statistical analyses of data over time (*t* = 0–120 min for in vivo microdialysis and *t* = 1–7 days for ethanol consumption), whereas one-way ANOVAs followed by Dunnet’s or Tukey’s post hoc tests were used for analyses of area under the curve (AUC) data. A probability value (*p*) less than 0.05 was considered statistically significant. In the microdialysis study, animals were classified as dopamine responders if dopamine was elevated more than 10% compared to baseline within the first hour after injection, in line with a previous dichotomy of animals that received glycine locally into the nAc (Molander and Soderpalm 2005a). In the ethanol consumption study, individual animals were classified as responders to treatment if their mean ethanol intake during days of treatment 1–7 was reduced by more than 10%, as compared to baseline. The limit was set to 10% based on the standard deviation of ethanol intake during baseline.

## Results

### Effects of systemic treatment with glycine on accumbal glycine and dopamine levels

**Fig. 2:**
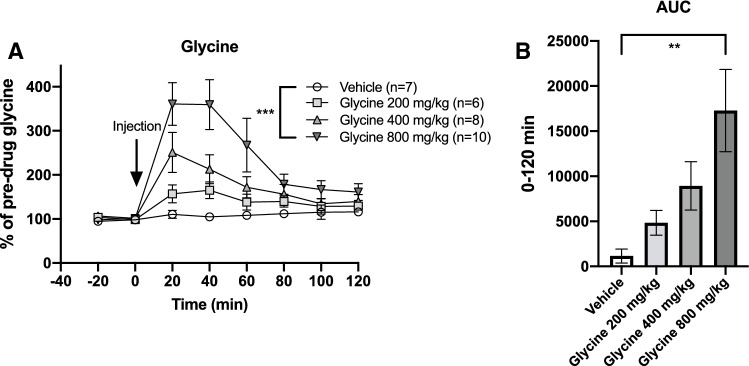
Systemic glycine elevates accumbal glycine levels. **a** Systemic administration (i.p.) of glycine significantly and in a dose-related manner increased accumbal glycine levels, as compared to vehicle treated controls. **b** Comparisons of AUCs also revealed a significant elevation of accumbal glycine levels. Shown are mean values ± SEM, *n* = number of rats, **p < .01, ***p < .001

**Fig. 3:**
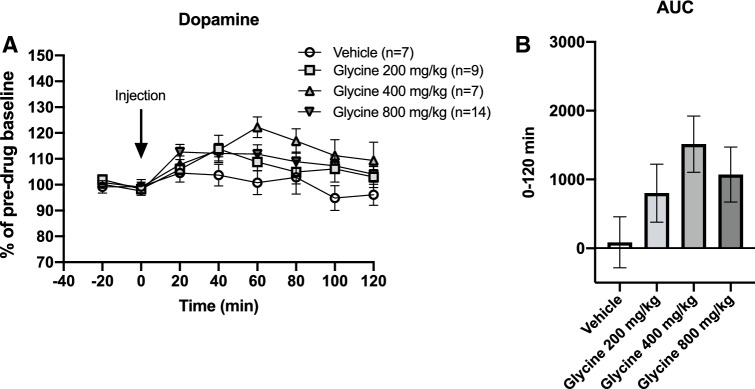
Effects of systemic glycine on accumbal dopamine levels. **a** After systemic administration of glycine (i.p.), a trend towards elevated accumbal dopamine levels was observed, especially following the intermediate dose. **b** Comparisons of AUCs failed to disclose a significant dopamine elevation. Shown are mean values ± SEM, *n* = number of rats

Basal glycine and dopamine concentrations in the dialysate were 1.15 ± 0.06 μM and 2.89 ± 0.25 nM, respectively. Systemic administration of glycine 200, 400 and 800 mg/kg i.p. increased accumbal glycine levels in a dose-related manner, two-way ANOVA_t=0–120_; treatment effect *F*_(3,27)_ = 7.27, *p* = 0.001, time effect *F*_(6,162)_ = 12.76, *p* < 0.001, interaction term *F*_(18,62)_ = 3.97, *p* < 0.001. Dunnet’s post hoc revealed a significant effect for glycine 800 mg/kg vs vehicle, *p* < 0.001 (Fig. 2a). Glycine peaks were achieved at either time-point 20′ or 40′ and reached levels of approximately 65, 150 and 260% for each dose, respectively. Comparisons of areas under the curve (AUC) also showed a dose-related, significant elevation of glycine, one-way ANOVA; *F*_(3,27)_ = 4.66, *p* = 0.009, Dunnet’s post-hoc glycine 800 mg/kg vs vehicle, *p* = 0.004 (Fig. 2b). Accumbal dopamine levels were also mildly elevated after treatment with systemic glycine, two-way-ANOVA_*t*=0–120_; treatment effect *F*_(3, 33)_ = 1.92, *p* = 0.145, time effect *F*_(6,198)_ = 10.02, *p* < 0.001, interaction term *F*_(18, 198)_ = 1.54, *p* = 0.080 (Fig. 3a). Comparisons of the AUCs did not reveal any significant alterations of accumbal dopamine levels (Fig. 3b).

### Effects of systemic treatment with glycine in dopamine responding animals

**Fig. 4:**
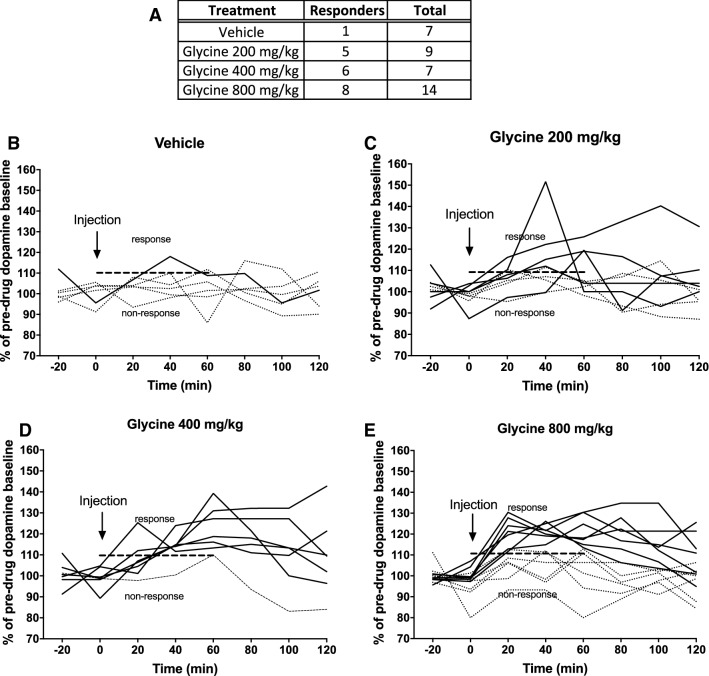
Distribution of dopamine responding animals. **a** Distribution of individual animals responding with an accumbal dopamine elevation after systemic treatment with glycine, by treatment group. A dopamine response was defined as an elevation of accumbal dopamine levels by more than 10% from baseline during the first hour after injection. **b**–**e** Each curve represents the accumbal dopamine response after treatment with vehicle, glycine 200 mg/kg, glycine 400 mg/kg or glycine 800 mg/kg i.p. for an individual animal. The solid line represents animals classified as responders whereas the dotted line represents animals classified as non-responders. Animals presenting a dopamine elevation above 10% compared to baseline during the first hour after injection were classified as dopamine responders. Among animals treated with 400 mg/kg glycine i.p., 6 out of 7 animals were classified as glycine responders, while a smaller proportion of glycine responders were identified among animals treated with glycine 200 mg/kg (5/9) or 800 mg/kg (8/14)

**Fig. 5:**
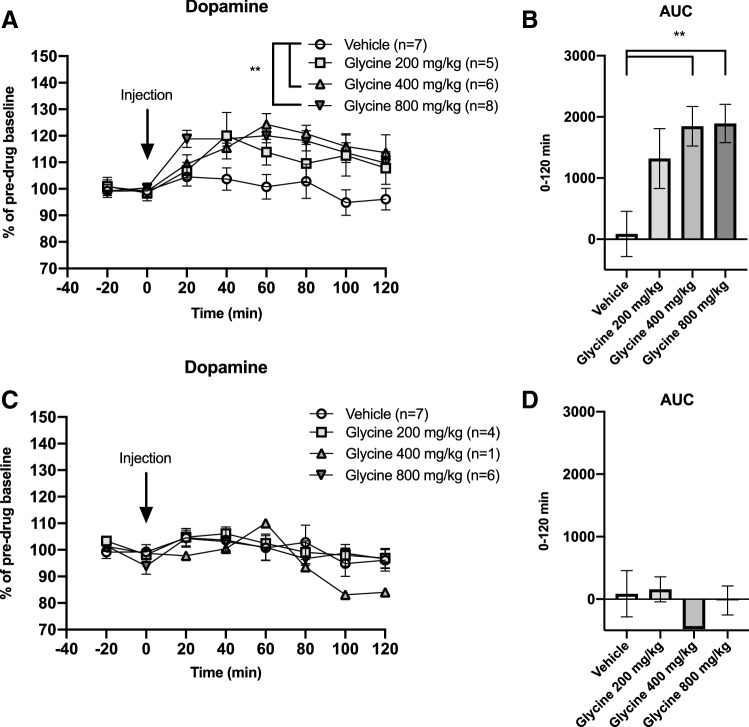
Systemic glycine elevates accumbal dopamine levels in a subpopulation of animals. **a** In glycine-treated animals defined as dopamine responders, systemic administration of glycine (i.p.) significantly increased accumbal dopamine levels, as compared to vehicle treated controls. In the dose-range studied, the effect did not appear to be dose-related. **b** AUCs differed significantly between animals treated with glycine i.p. and vehicle-treated controls. **c**, **d** Upon comparison of accumbal dopamine levels between glycine-treated animals defined as dopamine non-responders and vehicle-treated controls, no significant treatment effects could be revealed. Shown are mean values ± SEM, *n* = number of rats, **p < .01

In a next step, dopamine levels for each individual animal were scrutinized to examine whether only a subdivision of animals presented with a dopamine response, in accordance with observations made in animals perfused with glycine locally into the nAc (Molander and Soderpalm 2005a). Among animals treated with glycine 400 mg/kg i.p., six out of seven were classified as dopamine responders, i.e., presenting a dopamine elevation of more than 10%, whereas this proportion was smaller in animals treated with glycine 200 mg/kg (5/9) or 800 mg/kg (8/14) (Fig. 4). On the basis of this finding, results are also presented for dopamine responders alone. A two-way ANOVA revealed a significant elevation of accumbal dopamine; treatment effect *F*_(3,22)_ = 5.29, *p* = 0.007, time effect *F*_(6,132)_ = 9.86, *p* < 0.001, interaction term *F*_(18,132)_ = 1.71, *p* = 0.044. Dunnet’s post hoc revealed significant effects produced by the two highest doses, glycine 400 mg/kg vs vehicle *p* = 0.009 and glycine 800 mg/kg vs vehicle *p* = 0.005 (Fig. 5a). Likewise, the AUC was significantly increased in animals treated with the two highest doses; one-way-ANOVA *F*_(3,22)_ = 5.70, *p* = 0.005, Dunnet’s post hoc glycine 400 mg/kg vs vehicle *p* = 0.007, glycine 800 mg/kg vs vehicle *p* = 0.003 (Fig. 5b). In an attempt to characterize what distinguishes dopamine responders from non-responders, a further subdivision of animals was made based on whether the glycine peak occurred at time-point 20 or 40 min after injection, as depicted in Table 1.

**Table 1:** Timing of the glycine peak for dopamine responding and non-responding animals

	Responder DA	Non-responder DA	Total
Glycine peak 20′	8	2	10
Glycine peak 40′	3	5	8

Animals that received glycine i.p. demonstrated a peak value of glycine either at 20 or 40 min post-injection. The distribution of individual animals defined as dopamine responders appeared to be slightly skewed towards an early timing of the glycine peak

### Effects of systemic treatment with glycine on voluntary ethanol intake and preference

**Fig. 6:**
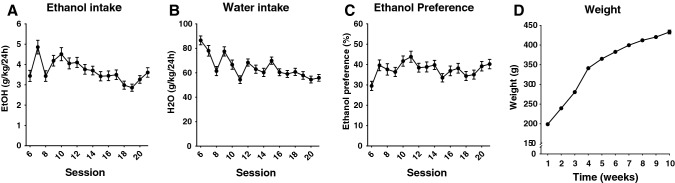
Screening for voluntary ethanol intake. **a**–**c** Ethanol intake, water, and ethanol preference remained on a stable level throughout the entire screening period. **d** Time course graph showing weight gain over time in all of the rats. Shown are mean values ± SEM

**Fig. 7:**
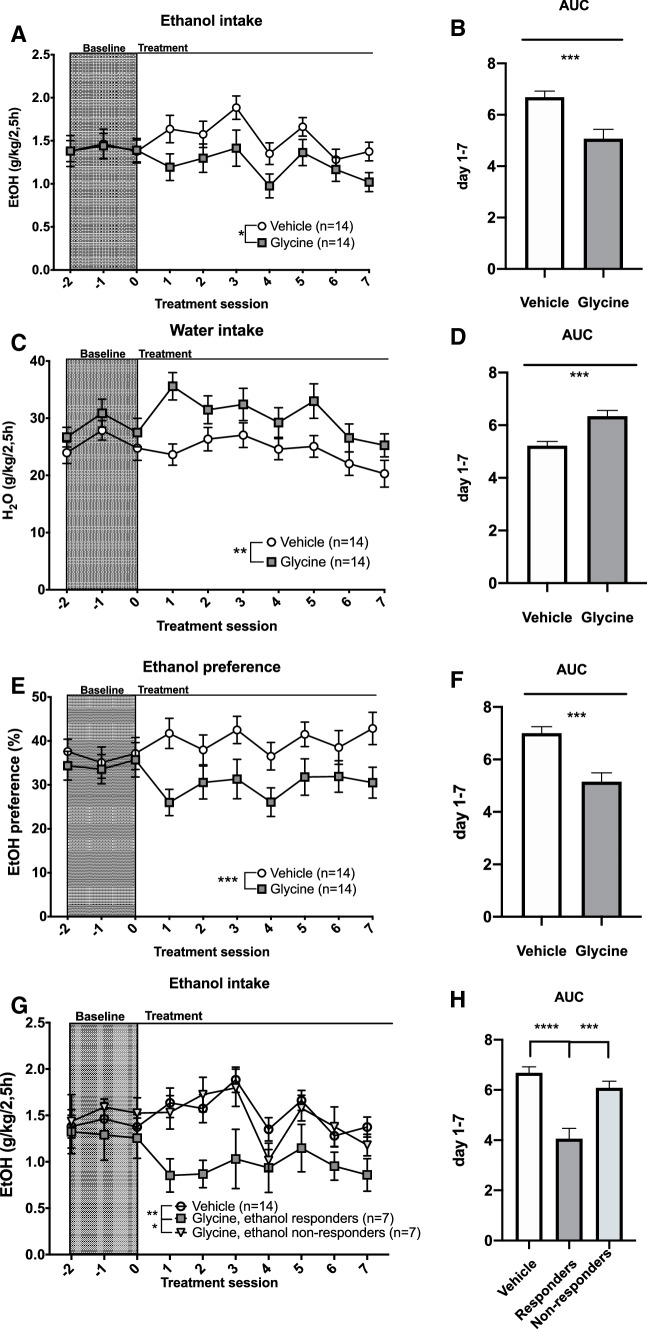
Treatment with glycine reduces ethanol intake. **a**, **b** Glycine (400 mg/kg, i.p.) reduced ethanol intake from the first day of treatment, as compared to vehicle-treated controls. **c**, **d** The glycine treated group increased its water intake from the first day of treatment. **e**, **f** Glycine treatment significantly reduced ethanol preference. **g**, **h** Upon subdividing glycine-treated animals into responders and non-responders, a significant reduction in ethanol intake was revealed for glycine-treated responding animals compared to both vehicle-treated and glycine-treated non-responding animals. Animals reducing their average ethanol intake during treatment day 1–7 with more than 10%, reflecting fluctuations at baseline, were classified as responders to treatment. Recordings were made during both baseline (gray) and active treatment periods. Shown are mean values ± SEM, *n* = number of rats, *p < 0.05, **p < .01, ***p < .001, ****p < 0.0001

In the second part of the study, the animals were screened for voluntary ethanol intake. Ethanol and water consumption remained relatively stable throughout the screening Fig. 6a–c). The rat weight was monitored once a week prior to and throughout the experiment, presenting stable weight-gain (Fig. 6d). After seven consecutive weeks of intermittent access to ethanol, the animals were placed on a limited access paradigm with access to water and ethanol for 2.5 h/day. Following 5 days of baseline consumption (including vehicle injections) the animals were divided into the two treatment groups [glycine (400 mg/kg) or vehicle (0.9% NaCl)]. The groups did not differ in ethanol intake during the baseline period, AUC, t test *p* = *0.988*. During the 7 days of active treatment, ethanol intake decreased in rats treated with glycine as compared to vehicle; two-way ANOVA_*t*=1–7_; treatment effect *F*_(1,26)_ = 5.65, *p* = 0.025, time effect *F*_(7,182)_ = 4.36, *p* < 0.001, interaction term *F*_(7,182)_ = 1.00, *p* = 0.433 (Fig. 7a); AUC, t test *p* = 0.001 (Fig. 7b). The glycine effect was evident on the first day of treatment. Simultaneously, water intake increased in the glycine treated group [treatment effect *F*_(1,26)_ = 4.77, *p* = 0.038, time effect *F*_(7,182)_ = 7.30, *p* < 0.001, interaction term *F*_(7,182)_ = 2.60, *p* = 0.014 (Fig. 7c); AUC, *t* test *p* < 0.001 (Fig. 7d)] and as a result, the ethanol preference decreased in the glycine treated group [treatment effect *F*_(1,26)_ = 7.06, *p* = 0.013, time effect *F*_(7,182)_ = 1.13, *p* = 0.345, interaction term *F*_(7,182)_ = 1.56, *p* = 0.149 (Fig. 7e); AUC, *t* test *p* < 0.001 (Fig. 7f)]. When comparing the average of ethanol intake during baseline and the average of ethanol intake treatment days 1–7, ethanol intake was slightly decreased by -0.22 g/kg/2.5 h in glycine-treated animals [one-way ANOVA*, F*_(7,91)_ = 2,232*, p* = 0.0385), whereas ethanol intake was slightly increased 0.16 g/kg/2.5 h in vehicle-treated animals (*F*_(7,91)_ = 3,137, *p* = 0.0052)]. To examine whether not only the dopamine response, but also the drinking response displayed a binary pattern consisting of responders and non-responders, animals were subdivided into two different groups based on whether their ethanol intake was reduced by more than 10%, reflecting fluctuations at baseline. In the vehicle-treated group, no animal was classified as a responder, whereas 7 out of 14 glycine-treated animals were responders (Table 2). A two-way ANOVA for this subdivision of animals revealed a significant alteration of ethanol intake; group effect *F*_(2,25)_ = 8.18, *p* = 0.002, time effect *F*_(7,175)_ = 3.85, *p* < 0.001, interaction term *F*_(14,175)_ = 1.37, *p* = 0.171. (Fig. 7g). Animals classified as responders presented a significant reduction in ethanol intake compared to both non-responders and vehicle-treated animals (Tukey’s post hoc responders vs non-responders *p* = 0.016, responders vs vehicle *p* = 0.002 (Fig. 7g). Correspondingly, AUC was significantly reduced in responding animals treated with glycine compared to non-responding animals treated with glycine or vehicle-treated animals; one-way ANOVA *F*_(2,25)_ = 20.02, *p* < 0.0001, responders vs vehicle *p* < 0.0001, responders vs non-responders *p* < 0.001 (Fig. 7h).

**Table 2:** Distribution of individual animals responding with decreased ethanol intake

Treatment	Responders	Total
Vehicle	0	14
Glycine	7	14

Animals were defined as responders if their average ethanol consumption treatment day 1–7 was reduced by more than 10%, compared to baseline. The limit was set to 10% based on the standard deviation of ethanol consumption during baseline sampling. Among animals treated with glycine 400 mg/kg i.p., half of the animals responded to treatment

## Discussion

This study shows that systemic administration of glycine increases glycine levels in nAc in a dose-related manner in the male Wistar rat, as measured by in vivo microdialysis. In a subpopulation of animals, also extracellular levels of dopamine were elevated in nAc, in line with previous findings with the GlyT-1-inhibitor Org 25935 (Lidö et al. 2009). Further, the study shows that systemic glycine treatment, in similarity with Org 25935, decreases ethanol intake and preference in a limited access two-bottle ethanol/water model in the rat (Molander et al. 2007). It has previously been demonstrated that (I) accumbal GlyR sustain basal dopamine levels and are required for ethanol-induced dopamine release in nAc (Molander and Soderpalm 2005a; b), (II) intracerebral (nAc) glycine and systemic glycine uptake inhibitors increase accumbal dopamine levels and attenuate the ethanol-induced dopamine elevation (Lidö et al. 2009, Molander and Söderpalm 2005b), and (III) intracerebral (nAc) glycine and systemic glycine uptake inhibitors reduce ethanol intake and preference in rats (Molander et al. 2005, 2007). The present results indicate that also systemic administration of glycine may be an alternative for modulating accumbal glycine and dopamine levels, and for reducing ethanol intake.

Glycine is one of the endogenous agonists to strychnine-sensitive GlyR, in turn one of the receptor populations through which ethanol is hypothesized to access the dopamine reward pathway (Molander and Söderpalm 2005b, Soderpalm et al. 2017, Spanagel 2009). Besides acting as a classic inhibitory neurotransmitter, glycine also activates the co-agonist site of the NMDA-glutamate receptor. This is the rational for its exploration in schizophrenia and other disorders hypothesized to be associated with impaired glutamatergic neurotransmission (Harvey and Yee 2013). Although high-dose adjunctive glycine has proven to ameliorate negative symptoms of schizophrenia, its efficacy is inconsistent, i.a. suggestive of insufficient brain penetration and high peripheral metabolism (Buchanan et al. 2007; Harvey and Yee 2013; Heresco-Levy et al. 1999). Focus has therefore shifted towards targeting glycine levels indirectly through GlyT inhibition. However, the present results in rats demonstrate a dose-related increase in striatal, extra-synaptic glycine levels, a slight dopamine elevation for a subpopulation of animals and decreased ethanol intake after systemic glycine treatment, all indicative of sufficient CNS penetrance.

Even though the present study in itself cannot exclude the possibility that these neurochemical and behavioral alterations stem from actions of glycine outside of the CNS, this possibility appears less likely. We are aware of no actions of peripheral glycine that may underlie such effects, whereas glycine applied locally into nAc increases accumbal dopamine via GlyR activation and reduces ethanol consumption in rats (Molander et al. 2005, Molander and Soderpalm 2005a). A tentative involvement of central NMDA receptors in the ethanol intake reducing effect should also be considered, since these receptors are well established targets for ethanol (Lovinger and Alvarez 2017). Arguing against NMDA receptor involvement is however the fact that NMDA receptor antagonists rather than agonists have been demonstrated to reduce both ethanol consumption in an alcohol relapse model in rats and ethanol conditioned place preference in mice (Vengeliene et al. 2005; Biała and Kotlińska 1999).

Although a trend for increments of accumbal dopamine after systemic glycine was observed, the results were not significant when regarding all animals as a homogenous group. In contrast to the dose-related response displayed by accumbal glycine, dopamine appeared to, at least in the dose-range investigated, exhibit an all or nothing response. When previously administrating glycine in perfusate concentrations of 100 micromolar or 1 mM via reversed microdialysis in the nAc, a similar phenomenon was noted (Molander and Soderpalm 2005a). Furthermore, only a subpopulation of the rats displayed a distinct dopamine response, i.e., a dopamine elevation by 10% or more compared to baseline during the first hour after injection, hence motivating a division between dopamine responders and dopamine non-responders (Molander and Soderpalm 2005a). Moreover, animals with dopamine elevations following bilateral perfusion of glycine in nAc reduced their ethanol intake, while no such effect was noted in dopamine non-responders (Molander et al. 2005). A binary response was also evident after systemic treatment with the GlyT1-inhibitor Org 25935, where a subpopulation of rats displayed a dopamine elevation and attenuation of the ethanol-induced dopamine response (Lidö et al. 2009). In the present study, glycine 400 or 800 mg/kg significantly elevated accumbal dopamine levels in the subpopulation of animals classified as dopamine responders (in 14 out of 21 rats), when compared to all vehicle-treated controls. Interestingly, also in the ethanol consumption study, the response was dichotomous, where half of the animals responded with reduced ethanol intake after systemic glycine treatment. It could thus be speculated that the subgroup of animals reducing ethanol intake after systemic glycine also were dopamine responders, as was evident following intracerebral (nAc) perfusion with glycine (Molander et al. 2005). However, to confirm this hypothesis, studies where microdialysis and measures of ethanol intake are performed simultaneously following systemic glycine are required.

Several mechanisms could contribute to the all or nothing dopamine response observed, both in the present study and in previously performed experiments manipulating central glycine levels. For instance, like other ligand-gated ion channels GlyRs may desensitize after prolonged or massive exposure to a ligand (Jones and Westbrook 1996, Lynch 2004), a phenomenon that may well have occurred when accumbal glycine was elevated by approx. 260% (time-point 20′) after 800 mg/kg of glycine, by far exceeding levels previously observed after a dose of Org 25935 reducing ethanol intake (Lidö et al. 2009). In the present study, the largest proportion of dopamine responders were observed after the intermediate dose (400 mg/kg) and the distribution of dopamine responders was slightly shifted towards animals displaying an early glycine peak. Whether a profile where glycine reaches a certain level sufficient to activate GlyRs early (at 20′ instead of 40′) but low enough to avoid rapid GlyR desensitization is required for dopamine activation needs to be further explored. However, it should be kept in mind that depending on concentrations, glycine could interfere with striatal targets other than GlyR, such as the NMDA receptor (see above) or the GABA_A_ receptor (Adermark et al. 2011; Shrivastava et al. 2011), which in turn also could affect dopamine levels and/or ethanol intake.

The propensity for a glycinergic compound to produce a sustained dopamine elevation may be related to its ability to reduce ethanol consumption. Compared to Org 25935 (Lidö et al. 2009), systemic glycine produced a slightly lower dopamine peak elevation with a later onset (approx. 25% at 60′ after glycine 400 mg/kg vs. 30% at 40′ after Org 25935 6 mg/kg), as well as a faster decline (approx. 15% at 100′ after glycine 400 mg/kg vs. 20% at 100′ after Org 25935 6 mg/kg), in dopamine responders. The shorter duration of the dopamine elevation in combination with administration of the drug 30 min prior to the 2.5 h drinking period may have contributed to a less pronounced, albeit significant reduction in ethanol intake, as compared to Org 25935 (Molander et al. 2007). Notably, in the present study, ethanol intake was slightly increased in vehicle treated animals, which may have exaggerated the difference in treatment outcome between vehicle and glycine (400 mg/kg). However, glycine reduced ethanol intake also when compared to baseline drinking and the concurrent increase in water intake and stable weight gain indicate that the observed effect did not stem from animal malaise but is suggestive of a specific ethanol intake reducing effect. Moreover, in previous studies, it was demonstrated that voluntary intake of comparable amounts of ethanol results in a significant elevation of dopamine in the nAc (Ericson et al. 1998), indicating that the ethanol consumption level here studied is of psychopharmacological relevance.

The modest dopamine response and reduction of ethanol intake in spite of a pronounced glycine elevation in accumbal dialysates raise the question of to what extent glycine passed the blood brain barrier (BBB) and reached the interstitial space. Inherent to the technique of in vivo microdialysis is a traumatic lesion when inserting the probe into nAc. The notion of whether the integrity of the BBB is maintained is both supported and contradicted by previous studies (de Lange et al. 1994; Groothuis et al. 1998). Thus, it could be argued that the dose-related increase of glycine in accumbal dialysates merely reflects direct passage into the probe through local damage to the BBB. On the other hand, the dopamine alterations observed argue that at least part of the elevated glycine derives from the interstitial space where neuronal activity indeed was affected.

Since systemic glycine elevated accumbal glycine and dopamine levels and slightly decreased ethanol consumption in a subpopulation of rats, a tentative future step may be to explore systemic glycine treatment for AUD. The underlying rationale being that glycine slightly elevates dopamine, thereby alleviating amotivation and anhedonia and simultaneously preventing ethanol-induced dopamine-elevation and reward (Soderpalm et al. 2017), it is advised that the design of a clinical trial allows simultaneous exposure to ethanol and glycine, in analogy with the design of the present study in rats. This requirement was not fulfilled in the negative clinical trial with Org 25935 (de Bejczy et al. 2014).

Some limitations of the present study should be pointed out. First, the drinking paradigm applied entailed limited access to alcohol and water for 2.5 h/day, meaning that the main incentive to drink may have been thirst. Also, we cannot be certain that rats were actively seeking for ethanol since the position of the ethanol and water bottle, respectively, was not switched. However, these possible constraints in the study design cannot fully explain the difference in ethanol preference between rats that received glycine or vehicle, which were equally fluid deprived and subjected to identical positioning of bottles. Second, the rats used in the in vivo microdialysis study, and most likely also the ones in the drinking study, were not alcohol dependent. Therefore, studies aimed at investigating if the present results apply also to alcohol-dependent subjects are desired.

To conclude, this is to our knowledge the first study of the effects of systemic treatment of glycine on accumbal glycine and dopamine levels and ethanol intake in the rat. Results from the present study in conjunction with previous findings give further support to the concept of modulating brain glycine levels as a tentative treatment strategy for AUD. However, further studies exploring the effects of systemic glycine in alcohol-dependent subjects as well as the nature underlying the dichotomous response to elevating glycine levels are warranted. Nevertheless, the present results indicate that the glycinergic system as a treatment target for AUD should not be prematurely dismissed.
